# Development of Label-Free Impedimetric Immunosensors for IKZF1 and IKZF3 Femtomolar Detection for Monitoring Multiple Myeloma Patients Treated with Lenalidomide

**DOI:** 10.1038/s41598-020-67241-w

**Published:** 2020-06-26

**Authors:** Haya Abdulkarim, Mohammed Zourob, Mohamed Siaj

**Affiliations:** 1 Department of Chemistry, Université du Québec à Montréal, Montréal, H3C 3P8 Quebec, Canada; 2 Department of Chemistry, Alfaisal University, Al Zahrawi Street, Al Maather, Al Takhassusi Road, Riyadh, 11533 Saudi Arabia; 3 King Faisal Specialist Hospital and Research Center, Zahrawi Street, Al Maather, Riyadh, 12713 Saudi Arabia

**Keywords:** Cancer stem cells, Immunochemistry

## Abstract

Lenalidomide is an immunomodulatory drug (IMiD) used to treat multiple myeloma (MM) patients. Lenalidomide destroys MM cells by inducing ubiquitination and the consequent degradation of Ikaros family zinc finger proteins 1 and 3 (IKZF1 and IKZF3). High expression of IKZF1 and IKZF3 in MM results in less sensitivity to lenalidomide treatment and possible cytotoxic effect. Therefore, detecting the expression of IKZF1 and IKZF3 proteins is of utmost importance in the treatment of MM. Here, we report the fabrication of a novel label-free electrochemical immunosensor for the rapid detection and quantification of IKZF1 and IKZF3 using electrochemical impedance spectroscopy (EIS). Gold electrodes were used to fabricate the immunosensors by immobilizing IKZF1 and IKZF3 specific antibodies using cysteamine and PDITC crosslinkers. The immunosensors were able to detect IKZF1 and IKZF3 protein levels with respective low detection limits of 0.68 and 0.97 pg/ml (11.8 and 16.7 fM). Furthermore, the immunosensors’ successful application in human serum and their high selectivity and sensitivity enables their possible application in other biofluids as simple point-of-care devices for monitoring multiple myeloma patients treated with lenalidomide, to prevent the drug’s cytotoxicity and minimize its side effects.

## Introduction

Multiple myeloma (MM) is the most common type of plasma cells cancer. It’s responsible for around tenth of all the haematological tumors, and 1.6% of all cancer cases^1^. Multiple myeloma is characterized by the irregular increase in the clonal plasma cells leading to the organ dysfunction^2^. Some of the symptoms associated with MM are nausea, weakness, and vomiting, others only manifest laboratory abnormalities like anemia and high monoclonal protein levels in serum or urine, and elevated clonal plasma cells in the bone marrow^1,2^. Multiple myeloma is known to be incurable, but major improvements happened in recent years, allowing younger patients to have a higher median survival life span^2^.

Generally, the discovery of immunomodulatory drugs (IMiDs) like lenalidomide and thalidomide, introduced some treatment options for MM^2,3^. However, Thalidomide was first used as a sedative and later for the treatment of MM, but then proven to cause miscarriages and birth defects, resulting in discontinuing its use^4^. A new less toxic IMiD called Lenalidomide (Revlimid) was later discovered^2^, and was proven to be a highly effective treatment for MM^5^. Lenalidomide’s minimal neurotoxicity allows it to be a long-term administrable drug for multiple myeloma patients^6^.

In Multiple myeloma, the continuous expression of Ikaros family zinc finger protein 1 (IKZF1) and Ikaros family zinc finger protein 3 (IKZF3) is essential for the proliferation and survival of the myeloma cells^5^. IKZF1 and IKZF3 are transcriptional factors that are important in the differentiation of lymphocytes, but their post transcriptional regulation is still poorly studied^3^. Lenalidomide kills multiple myeloma cells by inducing the IKZF1 and IKZF3 ubiquitination, causing the proteasomal degradation of both IKZF1 and IKZF3 B-cell transcription factors^5^. Krönke *et al*.^7^ suggested that the high expression of IKZF1 and IKZF3 in MM cells resulted in less sensitivity to lenalidomide treatment. The study also described the selective degradation of IKZF1 and IKZF3 caused by Lenalidomide in MM, and the importance of their loss during the therapeutic activity of Lenalidomide^8,9^. Several hours after treatment with lenalidomide, IKZF1 and IKZF3 get down regulated and degraded rapidly^10^.

Several immunoassays have been employed previously, including immunohistochemistry technique which was used to quantify IKZF1 and IKZF3, but it only permitted the semi-quantification of proteins and interpretation of the results require experienced pathologists^11^. Flow cytometry was also used^10^ for the analysis of IKZF1, as well as immunoblotting for IKZF1 and IKZF3 after the treatment with lenalidomide for MM patients^10^. Enzyme-linked immunosorbent assay (ELISA) and western blot (WB)^11^ are amongst other methods used. However both are time consuming, involve labeling with enzymes or tags^12^, and require trained staff and centralized labs to be performed^13^ and relatively large sample quantities.

Nowadays, more research is being conducted on simpler, more rapid, and cost-effective analytical assays such as biosensors and lab-on-a-chip (LOC) devices. More specifically, electrochemical biosensors show great promise due to their significant lower cost, minimization of the sample volume used, and their high throughput screening^14^, therefore they’re applicable as point-of-care testing (POCT) devices. To the best of our knowledge, this is the first report for the development of electrochemical biosensors for the detection and quantification of IKZF1 and IKZF3. Therefore, in this work we report the fabrication of new label-free impedimetric immunosensors for the detection of IKZF1 and IKZF3 at the femtomolar levels for MM patients given lenalidomide treatment. The immunosensors were fabricated by immobilizing IKZF1 and IKZF3 specific antibodies on classic gold electrodes and testing them further with different concentrations of the proteins. The method used was electrochemical impedance spectroscopy (EIS) which is known to be a non-destructive technique done in the presence of a redox couple also referred to as faradaic impedance measurements^13,15^. The detection was achieved by measuring the change in the charge transfer resistance as proteins bind to their respective immobilized antibodies. These label-free impedimetric immunosensors can be possibly useful for monitoring the response to lenalidomide in MM patients by detecting IKZF1 and IKZF3, and maybe later applied for monitoring different immunomodulators or therapeutic agents.

## Results and discussion

### Fabrication of the IKZF1& IKZF3 immunosensors

**Scheme 1:**
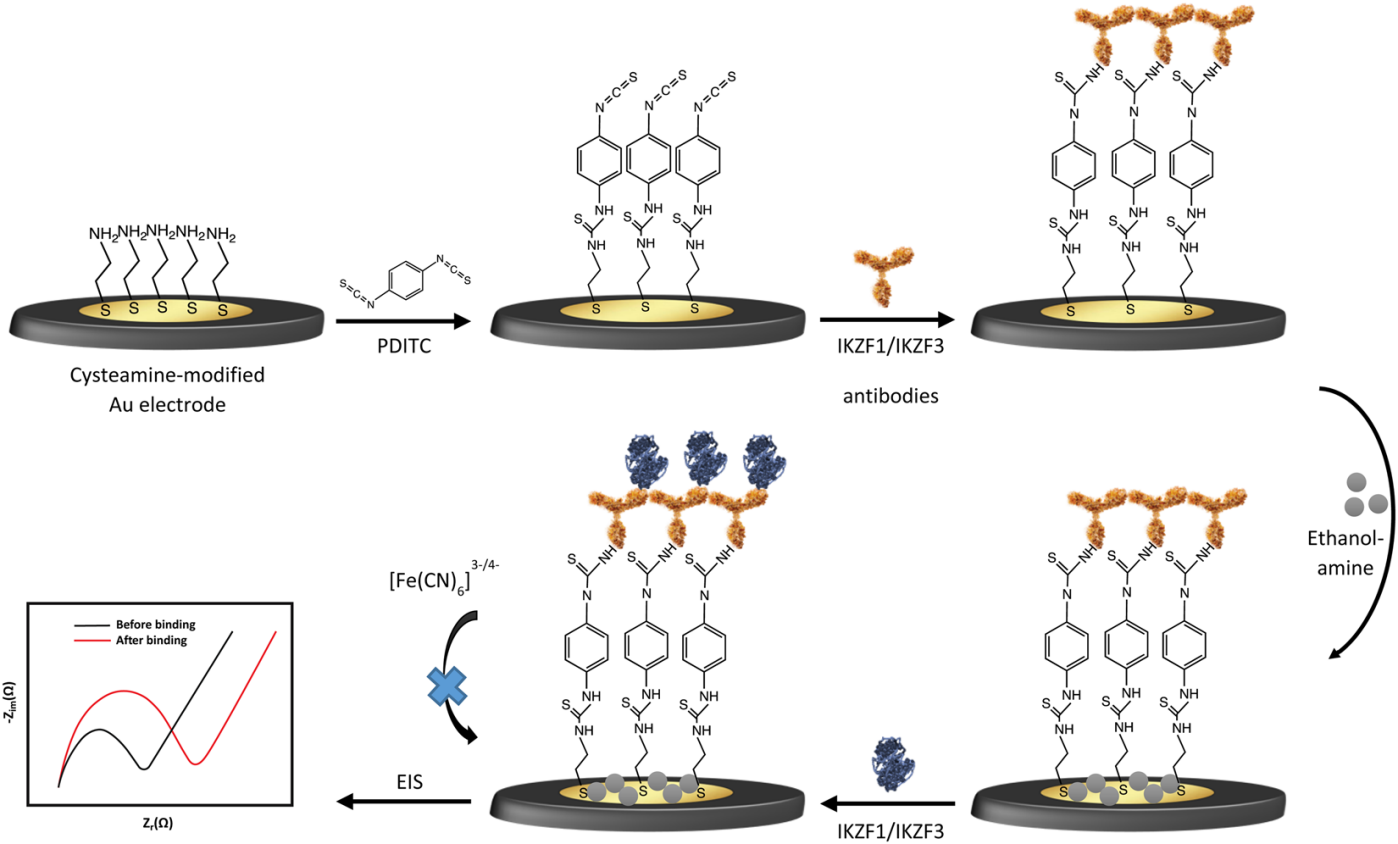
Schematic diagram of the fabrication steps and detection of the IKZF1 and IKZF3 immunosensors.

The immunosensors were fabricated by the immobilization of IKZF1 and IKZF3 specific antibodies on the surface of gold electrodes (Scheme 1.). For immobilization, standard gold electrodes were first modified with cysteamine and PDITC as a linker. Cysteamine was first incubated on the surface to form a self-assembled monolayer (SAM) containing terminal amine groups. PDITC was then used on cysteamine modified electrodes as a cross-linker. The formation of the thiourea bonds allows the thiocyanate groups from the PDITC to react with amine groups of the antibodies, and the other thiocyanate group would react with the amine groups present on the gold surface^15^. Finally, ethanolamine was used to block the unreactive thiocyanate groups and minimize the non-specific adsorption that might generate false signals when later applying the immunosensor. The immunosensor’s stability was also tested after storing it in PBS buffer pH 7.4 in 4 °C for 2 weeks with a response change of 2.8% and 1.9% for IKZF1 and IKZF3 immunosensors, respectively.

### Electrochemical characterization of the immunosensors

**Figure 1:**
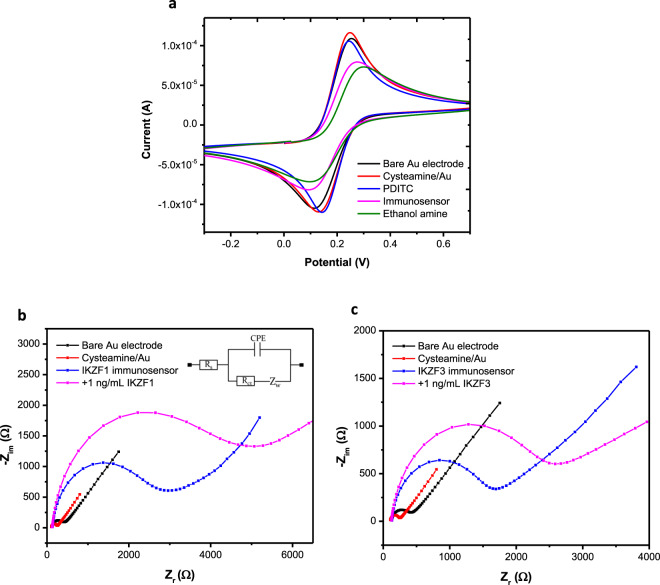
Cyclic voltammetry peaks for the characterization of the fabrication step of the immunosensors (**a**). Nyquist plots for the fabrication of IKZF1 immunosensor (**b**) and IKZF3 immunosensor (**c**). Figure 2b shows the modified Randles equivalent circuit used to fit all the EIS Nyquist plots in this work. CV was measured at scan rate of 100 mV/s, and EIS measurements were all done at 10 KHz to 0.1 Hz frequency range. Electrochemical measurements were done in ferro/ferricyanide redox couple solution in PBS pH 7.4.

EIS and CV were used to electrochemically characterize the gold electrode surface for each step of the functionalization of the IKZF1 and IKZF3 immunosensors. Both were recorded for each fabrication step of the two immunosensors in ferro/ferricyanide redox couple solution. For CV (Fig. 1a.), a sharp reversible peak was first observed for the clean gold electrode, exhibiting a peak-to-peak separation (ΔE). After functionalization with cysteamine, there was a slight decrease in the ΔE indicating the enhancement of the electron transfer due to the positive charge of the amine groups. But the addition of the PDITC and its reaction with cysteamine caused an increase in ΔE as the peak current decreased, that’s attributed to the negative charge of the composed terminal isothiocyanate groups which inhibited the electron transfer by revolting the redox anions^15^. The immobilization of the antibodies caused the ΔE to further increase and the electron transfer to decrease due to the blockage caused by the bulky sized antibodies.

For each fabrication step, EIS was measured as shown in (Fig. 1b,c.). Nyquist plots were obtained and fitted using modified Randles equivalent circuit consisting of solution resistance (R_S_), charge transfer resistance (R_ct_), constant phase element (CPE), and Warburg impedance (Z_W_). Nyquist plots obtained for each step consist of a semicircle indicating the charge transfer process, followed by a straight line indicating the diffusion of redox molecules. For the bare gold electrode, a typical small semicircle with a straight line was observed. After the functionalization of the surface with cysteamine, the semicircle diminished indicating a lower charge transfer resistance. After the functionalization with the crosslinker PDITC and the immobilization of the antibodies, the semicircle broadened significantly implying the slower electron transfer rate and higher electron transfer resistance. The semicircle will only further increase as each immunosensor is incubated with a higher concentration of its respective proteins, due to the proteins’ large size and their increasing blockage of the electrode surface, demonstrating the fundament of this immunosensor’s detection method.

### Binding experiments of the immunosensors with their specific proteins and binding time optimization

**Figure 2:**
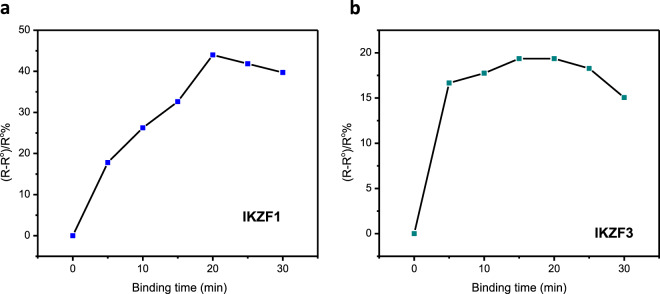
Plot of the immunosensor response versus the binding time in minutes of the immunosensors with their specific proteins for IKZF1 (**a**) and IKZF3 (**b**).

The binding time of antibodies to their antigens is important to ensure the highest signal response in the shortest time. In order to optimize this parameter for IKZF1 and IKZF3 immunosensors, both were incubated with 0.25 ng/ml of their respective analytes diluted in PBS (pH 7.4), then EIS plots were obtained at different periods (from 5 to 30 minutes). The percentage of change in the resistance before and after binding was used to calculate the sensor response as the following ((R-R°)/R° %), where R represents the resistance of the sensor after binding, and R° is the resistance before binding. Figure 2a,b show the responses of IKZF1 and IKZF3 immunosensors at different times. It is clear that the response was increasing until 20 minutes, after which no apparent increase was shown. The highest signal was obtained for IKZF1 immunosensor at 20 minutes, and between 15–20 minutes for IKZF3 immunosensor, indicating that 20 minutes is the shortest and optimal time to obtain the highest binding signal.

### Dose-response of IKZF1 & IKZF3 Immunosensors

**Figure 3:**
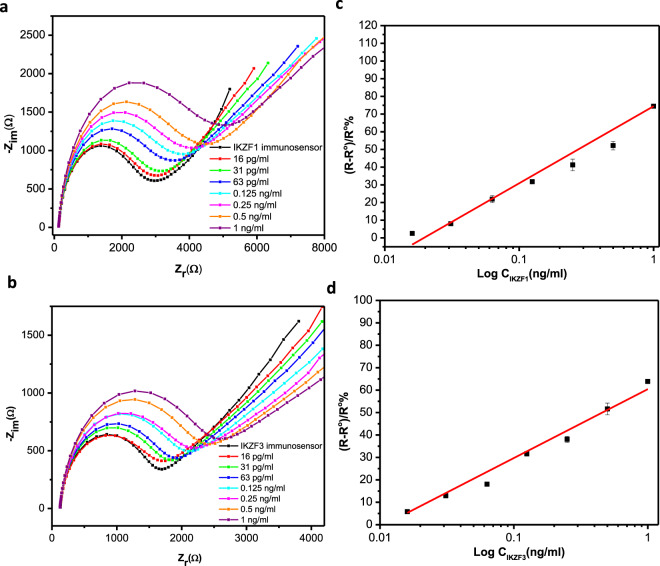
Plot of the immunosensor response versus the binding time in minutes of the immunosensors with their specific proteins for IKZF1 (**a**) and IKZF3 (**b**). EIS Nyquist plots after binding with different concentrations of IKZF1 (**a**) and IKZF3 (**b**), and the calibrations curves for IKZF1 (**c**) and IKZF3 (**d**) plotting the logarithm of concentrations in ng/ml versus the immunosensor’s response. Error bars were calculated as the SD of duplicate measurements.

The immunosensors were incubated in different concentrations ranging from 16 pg/ml to 1 ng/ml for both IKZF1 and IKZF3, and EIS was recorded for each concentration to test the analytical range of response. In (Fig. 3a,b.), Nyquist plots are shown for both immunosensors, for concentrations: 0, 0.016, 0.031, 0.063, 0.125, 0.25, 0.5, and 1 ng/ml. The consistent enlargement of the semicircle diameter is due to the increase of the charge transfer resistance caused by the increased concentration of proteins binding to the immunosensor. The data for both immunosensors were plotted into two calibration curves as shown in (Fig. 3c,d.), where the log of the protein concentration was plotted against the immunosensor response signal.

The linear regression equations for the immunosensors were: (R-R°)/R° % = 74.4 + 43.5 log C [ng/ml], R = 0.997 for IKZF1, and (R-R°)/R^o^ % = 60.4 + 30.6 log C [ng/ml], R = 0.985 for IKZF3. To confirm the high accuracy of the immunosensors, EIS plots were taken several times and the standard deviations of two measurements were demonstrated as error bars (RSD% < 4%). Both curves showed a linear response from 16 pg/ml to 1 ng/ml, and LOD were calculated to be 0.68 pg/ml (11.8 fM) for IKZF1, and 0.97 pg/ml (16.7 fM) for IKZF3 respectively, signifying the high sensitivity of the sensors at fM levels. However, the LOD of the ELISA kits were reported to be 31.25 pg/ml and 25 pg/ml for IKZF1 and IKZF3, respectively. ELISA has an Inter-Assay and Intra-Assay CV% for kits of <8% and <10%, respectively, as indicated by the manufacturer, whereas our sensors indicated <5% and <3% showing better reproducibility. The fabricated sensors are shown to be superior to ELISA by being label-free, cost-effective, faster, and have high potential to be applied as a point-of-care near bead-site or in-field applications.

### Cross reactivity testing of IKZF1 & IKZF3 immunosensors

**Figure 4:**
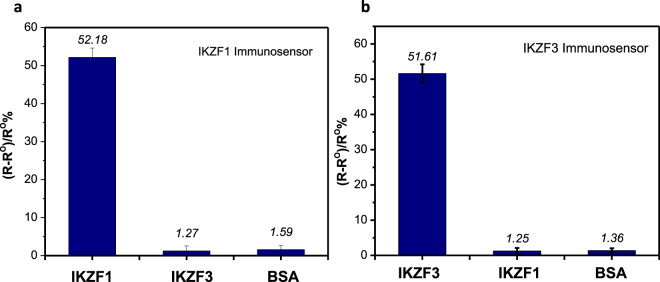
Cross reactivity experiments for the response of IKZF1 immunosensor against 0.5 ng/ml IKZF3 and 1% BSA (**a**), and IKZF3 immunosensor’s response against 0.5 ng/ml IKZF1 and 1% BSA (**b**), all in PBS buffer pH 7.4.

The selectivity of the immunosensors towards their specific proteins were examined against the other protein and against BSA. IKZF1 immunosensor was incubated in 0.5 ng/ml IKZF3 proteins and BSA, while IKZF3 immunosensor was incubated in 0.5 ng/ml IKZF1 protein and BSA. A drastic difference between the response of the sensors was observed towards their specific analyte in comparison with the non-specific proteins and BSA. Figure 4a,b show that IKZF1 immunosensor indicated a 52.18% response corresponding to its specific protein, in comparison to 1.27% response to IKZF3 proteins and 1.59% for BSA. On the other hand, IKZF3 immunosensor showed a 51.61% response corresponding to its specific protein, in comparison to 1.25% response to IKZF1 proteins and 1.36% for BSA. The results above demonstrate the high selectivity and very low cross reactivity of the developed immunosensors.

### Initial application of immunosensors in spiked serum samples

**Figure 5:**
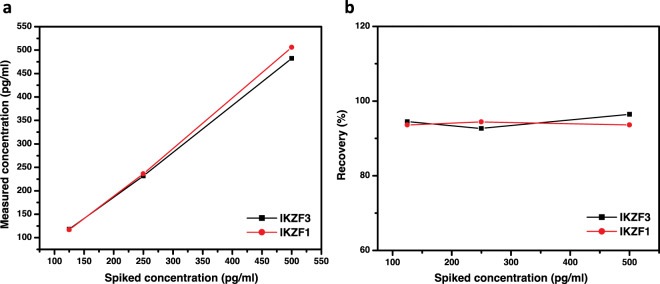
Spiked serum samples of IKZF1 and IKZF3 of different concentration 125, 250 and 500 pg/ml were plotted against the measured concentrations of the samples (**a**) their Recovery % (**b**).

To test the immunosensors and their ability to be applied in biological samples, they were applied on 1:8 diluted human serum spiked with 0.5 ng/ml IKZF1 and IKZF3. After measuring the concentrations, the relative standard deviation (RSD %) was calculated for the two measurements (Table 1). To further confirm the range of linearity, two more concentrations were tested by spiking serum with 0.125 and 0.25 ng/ml and plotting them against the measured concentrations (Fig. 5a.). Spiked concentrations were then plotted against their recovery % that ranges between 92.6% and 101.2% for IKZF1/3 at different concentrations (Fig. 5b.). The resulting recovery % eliminate the notable serum matrix effect on the immunosensor, indicating the sensors’ good precision and possible applicability in other biological samples suitable for IKZF1/3 detection.

**Table 1:** Two human serum samples (diluted 1:8) one spiked with 0.5 ng/ml of IKZF1 and the other with 0.5 ng/ml IKZF3, recovery %, and the relative standard deviation of duplicate measurements.

	Spiked concentrations *(pg/ml)*	Measured concentrations *(pg/ml)*	Recovery (%)	RSD (%)
IKZF1	500	482.3	96.5	13.2
IKZF3	500	506	101.2	2.8

## Experimental

### Materials and reagents

Cysteamine hydrochloride, Sulfuric acid, 1,4-phenylene diisothiocyanate (PDITC), pyridine, potassium ferrocyanide (K_4_Fe(CN)_6_), potassium ferricyanide (K_3_Fe(CN)_6_), ethanol amine, sodium chloride, N-N Dimethylformamide (DMF), hydrogen peroxide, acetonitrile, ethanol, Bovine serum albumin (BSA), phosphate buffered saline (PBS pH 7.4) tablets, and Human Serum (from male AB clotted whole blood) were obtained from Sigma-Aldrich (St. Louis, MO, USA). MicroPolish Alumina 0.05, 0.3, and 1.0 µm and polishing pads were bought from Buehler (Lake Bluff, IL, USA). Human DNA-binding Protein Ikaros (IKZF1) ELISA kit, and Human Zinc Finger Protein Aiolos (IKZF3) ELISA kit were purchased from Mybiosource (San Diego, CA, USA). PBS (pH 8.5) was used for the preparation of IKZF1 and IKZF3 antibody solutions. Highly pure water was obtained after Milli-Q plus treatment (Millipore, Billerica, MA, USA) then used for the preparation of solutions.

### Instrumentation

All electrochemical measurements for both cyclic voltammetry (CV) and Electrochemical Impedance Spectroscopy (EIS) were done using AUTOLAB PGSTAT302N potentiostat (Metrohm, Netherlands). The potentiostat contains a frequency response analyzer (FRA) connected to a computer run by NOVA 1.9 software. A classical electrochemical cell setup with three electrodes (counter, reference, and working) was used in all electrochemical experiments. The reference electrode is an Ag/AgCl (1 M KCL), the counter electrode is a platinum wire, and the working electrode is a standard gold electrode.

## Methods

### Fabrication of IKZF1 and IKZF3 immunosensors

The surface of bare gold working electrodes was first polished until a mirror finish was obtained using 1.0, 0.3 and 0.05 μm Alumina slurries on polishing pads for 5 minutes each. Polished electrodes were then cleaned with piranha solution 1:3 v/v of hydrogen peroxide (H_2_O_2_) and concentrated sulfuric acid (H_2_SO_4_), respectively, for 2 minutes at room temperature, then rinsed with deionized water followed by sonication in absolute ethanol for 2 minutes. The gold electrodes were then electrochemically cleaned by cycling the potential (vs. Ag/AgCl) between −0.2 and 1.6 V at 100 mV/s in 0.1 M H_2_SO_4_ for 30 cycles, then washed with deionized water. Subsequently, self-assembled monolayers were formed on the electrode surfaces, by incubating them in 10 mM cysteamine hydrochloride for 2 hours in room temperature. The modified electrodes were then washed with absolute ethanol to eliminate excess cysteamine, then incubated in 10 mM PDITC solution for 2 hours. PDITC was prepared as a mixture of 1:9 v/v pyridine and DMF. Electrodes were later washed with DMF and ethanol extensively. The modified electrodes were then incubated in a solution of PBS (pH 8.5) containing 1 µg/ml of IKZF1 or IKZF3 antibodies for 2 hours at room temperature, then washed with PBS (pH 7.4) to eliminate unbound antibodies from the surface. Finally, the electrodes were blocked by incubating them for 30 minutes in 0.1 M ethanol amine. They were washed with PBS (pH 7.4) afterwards, and directly used for detecting IKZF1 and IKZF3, or stored in PBS at 4°C.

### Immunosensor detection experiments

Electrodes immobilized with IKZF1 and IKZF3 were incubated for 20 minutes in tubes containing 100 µL of IKZF1 and IKZF3 protein solutions diluted in PBS (pH 7.4) at different concentrations (from 16 pg/ml to 1 ng/ml), electrodes were then washed with PBS buffer after each incubation. EIS and CV were the electrochemical measurements utilized for detecting.

### Electrochemical measurements

5 mM of Ferri/Ferrocyanide in PBS (pH 7.4) solution was used in all electrochemical measurements for EIS and CV. CV measurements were done by cycling the potential between −0.2 to 0.5 V at a scan rate of 100 mV/s. Whereas EIS measurements were recorded at a frequency of 10 kHz to 0.1 Hz, AC voltage of 10 mV and DC potential of 0.2 V. Nova 1.9 was used to fit all Impedance data displayed as Nyquist plots.

### Selectivity experiments

IKZF1 and IKZF3 immunosensors were tested for cross reactivity between the two proteins and for Bovine serum albumin (BSA). IKZF1 immunosensors were incubated in 100 µL of 0.5 ng/ml of IKZF3 or 1% BSA in PBS (pH 7.4). Similarly, IKZF3 immunosensors were incubated in IKZF1 and BSA for 20 minutes at room temperature.

### Application of the immunosensors in spiked serum samples

The immunosensors were applied on spiked serum samples, in order to examine the applicability of the sensor in real biological samples. Human serum samples (Sigma-Aldrich) were diluted 1:8 in PBS buffer (pH 7.4) and spiked with 0.125, 0.25 and 0.5 ng/ml of IKZF1 or IKZF3, then incubated on the electrode surface for 20 minutes at room temperature. Electrodes were then washed with PBS buffer and measured.

### ELISA experiments for IKZF1 and IKZF3 in serum

Duplicates of ELISA for IKZF1 and IKZF3 were performed according to the manufacturer’s protocol. First, the standards and spiked diluted serum samples were added to the antibody precoated wells and incubated for 2 hours at 37 °C. After washing, 100 μL of Biotin-conjugated secondary antibodies for 1 hour at 37 °C. Wells were washed and incubated for 1 hour with 100 μL of Horseradish Peroxidase (HRP) conjugated avidin at 37 °C. 90 μL of 3,3′,5,5′-Tetramethyl-benzidine (TMB) substrate solution were added to the wells and incubated for 15–30 minutes, then stopped by adding 50 μL of sulfuric acid stop solution. Readings were taken at 450 nm using a microplate reader.

## Conclusion

To summarize, new and simple label-free impedimetric immunosensors for the detection and quantification of IKZF1 and IKZF3 were fabricated. The rapid immunosensors showed very good sensitivity and selectivity against the other proteins, as well as good LOD of 11.8 and 16.7 fM (0.68 pg/ml and 0.97 pg/ml) for IKZF1 and IKZF3 immunosensors, respectively. The electrochemical immunosensors’ successful application in human serum opens the possibility of their future application in other biological samples taken from patients with MM treated with lenalidomide. Those biosensors could possibly work as point-of-care devices to monitor patient’s response and resistance to lenalidomide and treatment dosages required, to perhaps prevent cytotoxicity and reduce its side effects.
